# Patient survival after resection of skeletal metastases and endoprosthetic reconstruction: a nation-wide cohort study in a single oncological institution

**DOI:** 10.2478/raon-2025-0009

**Published:** 2025-03-19

**Authors:** Aljaz Mercun, David Martincic, Blaz Mavcic

**Affiliations:** Department of Orthopaedic Surgery, University Medical Centre Ljubljana, Ljubljana, Slovenia; Chair of Orthopaedics, Faculty of Medicine, University of Ljubljana, Ljubljana, Slovenia

**Keywords:** skeletal metastases, wide resection, endoprosthetic reconstruction, patient survival

## Abstract

**Background:**

The aim of this nation-wide 2009-2021 cohort study was to analyze postoperative survival of patients with resected appendicular skeletal metastases and endoprosthetic reconstruction in comparison to sarcoma patients and non-oncological reconstructions.

**Patients and methods:**

A single institution nation-wide cohort of 144 consecutive patients with tumor endoprosthetic reconstructions (32 resected metastases, 73 resected sarcomas, 39 non-oncological) were stratified into histopathological groups according to the 2013-SPRING prediction model. Their survival was analyzed with the Kaplan-Meier method and Cox regression.

**Results:**

The observed patient survival rates after wide resection of fast/moderate/slow growing metastases were 25/55/88% at 2 years and 10/30/83% at 5 years, while in sarcomas the observed survival rates were 80% at 2 years and 69% at 5 years. Estimated mean postoperative survival after resection of skeletal metastases was significantly shorter in comparison to sarcomas (4.6 years vs. 9.1 years, log-rank p < 0.001). Predictors of worse patient survival included higher age, pathologic fracture or >1 metastasis, diagnostic group fast-growing metastases and higher preoperative C-reactive protein (CRP).

**Conclusions:**

Wide resection and endoprosthetic reconstruction offer a reliable solution in selected patients with skeletal metastases. Higher age, fast-growing metastases (from bladder cancer, colorectal, hepatocellular, lung cancer, malignant melanoma, unknown origin), pathologic fracture or >1 metastasis and elevated CRP predict shorter patient survival and may represent a relative contraindication in this regard.

## Introduction

Endoprostheses are currently used as the prevalent limb-sparing surgical reconstruction option after wide tumor resections (curative intent for malignant tumours) of bone and cartilage in skeletally mature patients.^1–3^ Improvement of carcinoma patients’ survival with skeletal solitary/oligometastases in the last decade increased the number of metastatic resections and endoprosthetic recon-structions.^3,4^ Complex algorithms have been developed to help with the choice of the optimal surgical treatment option for skeletal metastases^5^, but the final decision and responsibility still lies with the appropriate tumor board. No nation-wide study so far has assessed patient survival after wide resection of skeletal solitary/oligometastases in comparison to sarcoma patients which is a similar surgical procedure. We decided to perform this cohort study with a single oncological decision-making institution (Oncological Institute) and a single department for wide resections of musculoskeletal tumors (University Medical Centre Ljubljana, Department of Orthopedic Surgery) where a single modular endoprosthetic system MUTARS® has been used for this purpose since 2009.^6^

The primary aim of this cohort study was to analyze postoperative survival of patients who underwent wide resection and endoprosthetic reconstruction of skeletal metastases in the Republic of Slovenia 2009-2021; to evaluate the impact of covariables (age, gender, histopathological diagnosis, pathologic fracture or > 1 metastasis, preoperative CRP / leukocyte count / haemoglobin / thrombocyte count) on this outcome and to compare them with sarcoma patients and non-onco-logical revision endoprosthetic patients operated in the same time period. Our secondary goal was to determine and compare implant removal rates between these patient groups, as all reconstructions in the observed period were performed with an identical modular tumor endoprosthetic system (MUTARS®).

## Patients and methods

The retrospective cohort consisted of patients who have undergone bone resection and reconstruction with a tumor endoprosthesis in the Republic of Slovenia between January 1st, 2009 and December 31st, 2021 at a single tertiary tumor center (Department of Orthopedic Surgery, University Medical Centre Ljubljana, Slovenia). This institution is the only department in the Republic of Slovenia to perform MUTARS® endoprosthetic system for tumor reconstructions used 2009-2021. The presented study group of 144 consecutive patients included cohort of resected bone metastases with endoprosthetic reconstruction in the selected observation period. No patients were excluded from the study. Patients were further stratified into five groups according to the 2013-SPRING survival prediction model:1) fast-growing metastases (bladder, colorectal, hepatocellular, lung, malignant melanoma, unknown, others), 2) moderate-growing metastases (prostate, renal), 3) slowgrowing metastases (breast, lymphoma, myeloma), 4) sarcomas and 5) non-oncological diagnoses (benign tumor resections, revision arthroplasty cases).^5^ All indications for wide resection, possible adjuvant radiotherapy or systemic therapies were confirmed by a single oncological decision-making institution (Institute of Oncology Ljubljana, Ljubljana, Slovenia). The following data was obtained for each patient included in this study: age, gender, histopathological diagnosis, anatomical localization of the resected tumor, date of tumor endoprosthesis implantation, number of detected metastases at the time of tumor resection, presence of pathologic fracture(s), date of possible subsequent implant removal, date of death (if applicable) and living status (alive/deceased) on October 1st, 2022. In the population of 32 metastatic patients (i.e. histopathological groups of fast-, moderate- and slow-growing metastases pooled together) we also analyzed the preoperative laboratory values of inflammation (CRP, leukocyte count, hemoglobin and platelets).

### Implants

The MUTARS® system (Modular Universal Tumor and Revision System; Implantcast, Buxtehude, Germany) was introduced in 1992 and has since been widely used in Europe and throughout the world in orthopaedic oncology as well as revision surgery.^1,7,8^ Many studies reported using MUTARS as revision endoprosthesis after failed primary total knee arthroplasty^7,9,10^, oncological pelvic and lover limb reconstruction^11–15^ and upper limb reconstruction.^16,17^ However, no study so far has evaluated nation-wide diagnosis-stratified patient survival after implantation of modular universal tumor and revision system (MUTARS®) or any other comparable modular tumor endoprosthetic system.

### Statistical analyses

Statistical data analysis was performed with Office 365 Excel (Microsoft Corp. Redmond, WA, USA) and SPSS Statistics 27.0 for Windows (IBM Corp, Armonk, NY, USA). Life tables of surviving patients 2 years and 5 years postoperatively were compared with the chi-square test. Estimated mean survival times after the index operation were computed with the Kaplan-Meier method and the differences between groups evaluated with the log-rank test. The impact of age, gender, histopathological group and oncological stage (i.e. presence of pathologic fracture or more than one metastasis) on postoperative patient survival was analyzed with the Cox regression model. In the subcohort of 32 metastatic patients (i.e., histopathological groups of fast-, moderate- and slowgrowing metastases pooled together), a separate Cox regression model was used to analyze the impact of preoperative laboratory values of inflammation (CRP, leukocyte count, hemoglobin and platelets) on postoperative patient survival.

### Ethical issues

The presented non-interventional observational retrospective study was approved by the National Medical Ethics Committee of the Republic of Slovenia (case No. 0120-486/2017/4). There was no funding and no conflict of interest.

## Results

Between January 1st, 2009, and December 31st, 2021, a total of 144 MUTARS® reconstructions were performed (10 pelvises, 4 total femoral, 37 proximal femoral, 2 femoral diaphyseal, 38 distal femoral, 21 revisions after primary total knee arthroplasty, 11 proximal tibial and 21 proximal humeral replacements). The mean age at the time of reconstruction was 49.9 ± 20.4 years. When patients were stratified into five groups based on the histopathological diagnosis of the resected tumor^5^, there were considerable differences in their mean age and percentage of patients in advanced oncological stage (pathologic fracture or >1 metastasis) at the time of the index operation (Table 1).

**TABLE 1.** Demographic characteristics, oncological stage and observed survival of patients stratified according to the histopathological diagnosis according to the 2013-SPRING survival prediction model

	Fast-growing metastases	Moderate-growing metastases	Slow-growing metastases	Sarcomas	Non-oncological patients
No. of subjects	12	11	9	73	39
Mean age [years]	63 ± 14	65 ± 10	64 ± 12	42 ± 21	53 ± 19
Gender [Female/Male]	6 / 6	2 / 9	8 / 1	39 / 34	20 / 19
Percentage of patients with pathological fracture or > 1 metastasis	75%	73%	44%	18%	0%
Patients alive 2 years after the operation ^†^	25%	55%	88%	80%	100%
Patients alive 5 years after the operation ^‡^	10%	30%	83%	69%	97%
Implant removed within 2 years after the operation ^†^	0%	9%	13%	9%	11%
Implant removed within 5 years after the operation ^‡^	10%	10%	17%	18%	19%

† in the subcohort of 139 patients with minimum 2 years of postoperative follow-up

‡ in the subcohort of 108 patients with minimum 5 years of postoperative follow-up

### Patient survival

Observed percentage of surviving patients after resection of fast- and moderate-growing metastases (2-year survival 25% and 55%, 5-year survival 10% and 30%, respectively) was considerably lower in comparison to slow growing metastases (2-year survival 88%, 5-year survival 83%) or sarcoma patients (2-year survival 80%, 5-year survival 69%) (Table 1) with statistical significance at both 2 years (p < 0.01) and 5 years of follow-up (p < 0.01).

**FIGURE 1.**
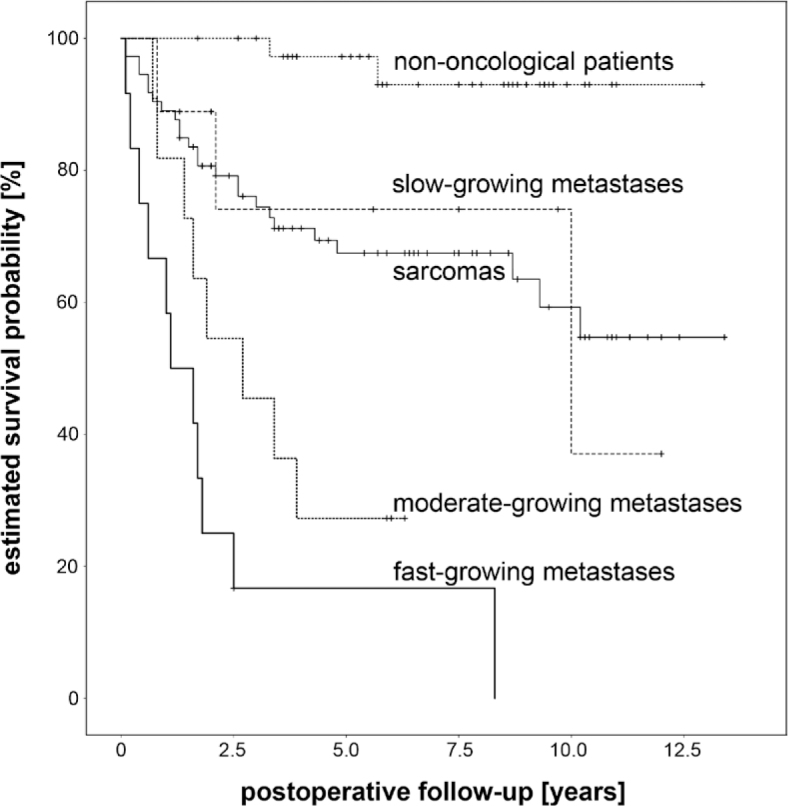
The Kaplan-Meier estimated mean survival time after surgical resection.

The Kaplan-Meier estimated mean survival time (Figure 1) after the index operation was 2.3 years for fast-growing metastases, 3.2 years for moderate-growing metastases, 8.5 for slow-growing metastases. When pooled together, the estimated mean postoperative survival of all resected skeletal metastases was significantly shorter in comparison to sarcomas (4.6 years vs. 9.1 years, logrank p < 0.001). In the Cox multivariate regression of postoperative patient survival after wide tumor resection, statistically significant predictors of worse outcome included higher age (hazard ratio for every additional year 1.021, p = 0.031), pathologic fracture or >1 metastasis (hazard ratio 2.809, p = 0.003) and histopathological group of fast-growing metastases (hazard ratio 5.522, p = 0.011), while the trend of shorter survival in moderate-growing metastases and sarcoma patients in comparison to the reference group of slow-growing metastases was not statistically significant (Table 2). Additionally, in the subcohort analysis of 32 metastatic patients shown in Table 3 (i.e. histopathological groups of fast, moderate- and slow-growing metastases pooled together), elevated CRP concentration was the only significant laboratory parameter predicting shorter survival (hazard ratio 1.018 for increase of 1 mg/L, p = 0.021).

**TABLE 2.** Cox regression analysis of patient survival for the cohort of 144 patients with tumor endoprosthetic reconstructions, stratified into histopathological groups according to the 2013-SPRING survival prediction model

	B	SE	Exp(B)	95 % CI for Exp(B)		p-value
				Lower	Upper	
Age [per year]	0.021	0.010	1.021	1.002	1.041	0.031*
Gender [male = 1]	-0.410	0.304	0.664	0.366	1.205	0.178
Slow-growing-mets (reference)						0.002*
Fast-growing mets	1.709	0.674	5.522	1.473	20.703	0.011*
Moderate-growing mets	0.853	0.703	2.347	0.592	9.308	0.225
Sarcomas	0.831	0.661	2.296	0.629	8.378	0.208
Non-oncological patients	-1.315	0.964	0.269	0.041	1.776	0.173
Pathological fx or > 1 mets	1.032	0.350	2.809	1.414	5.587	0.003*

1 B = Cox coefficient; CI = confidence interval; Exp(B) = risk for a revision; fx = fracture; mets = metastases; SE = standard error

* Omnibus test of model coefficients p < 0.001. Statistically significant p-values are marked with an asterisk

**TABLE 3.** Cox regression analysis of patient survival for the subcohort of 32 patients with metastases, stratified into histopathological groups according to the 2013-SPRING survival prediction model

	B	SE	Exp(B)	95 % CI for Exp(B)		p-value
				Lower	Upper	
Age [per year]	0.039	0.037	1.040	0.967	1.119	0.293
Gender [male = 1]	0.235	0.622	1.265	0.373	4.285	0.706
Slow-growing-mets (reference)						0.022*
Fast-growing mets	1.859	0.898	6.419	1.105	37.294	0.038*
Moderate-growing mets	0.436	1.014	1.547	0.212	11.289	0.667
Pathological fx or > 1 mets	-0.683	0.725	0.505	0.122	2.093	0.346
C-reactive protein [mg/L]	0.018	0.008	1.018	1.003	1.034	0.021*
Leukocyte count [10^9^/L]	0.096	0.081	1.100	0.940	1.289	0.234
Hemoglobin [g/L]	0.027	0.029	1.027	0.971	1.087	0.348
Thrombocyte count [10^9^/L]	0.000	0.005	1.000	0.989	1.011	0.980

1 B = Cox coefficient, CI = confidence interval; Exp(B) = risk for a revision; fx = fracture; SE = standard error, omnibus test of model coefficients p = 0.001. Statistically significant p-values

* are marked with an asterisk

### Implant survival

The cumulative number of implants requiring subsequent surgical removal for any reason during the follow-up was 24 (16.7%) out of the entire cohort of 144 MUTARS® endoprostheses. At 2/5 years after the operation, implants had to be removed in 6/8% of patients with skeletal metastases, 9/18% with sarcomas and 11/19% of non-oncological patients (Table 1). The Cox regression analysis of implant survival until removal showed none of the input variables (age, gender, histopathological group, pathologic fracture or > 1 metastasis) significantly affected endoprosthesis removal.

## Discussion

Life expectancy of oncological patients with skeletal metastases of appendicular skeleton has been extensively studied in the past, but few reports assessed patient survival after wide resection of skeletal metastases in comparison to sarcoma patients. In this nation-wide cohort study, patients with resected skeletal metastases had significantly shorter estimated postoperative survival (2.3-8.5 years) in comparison to bone sarcomas (9.1 years) or non-oncological revisions, but it was long enough to justify endoprosthetic reconstruction. Higher age, metastases other than plasmacytoma/renal cell/breast carcinoma, pathologic fracture or >1 metastases and elevated CRP values were independent predictors of shorter postoperative patient survival.

While most articles focus mainly on survival of implants in treatment of primary bone tumors (2-year survival of 86%, 5-year survival of 70.5-78.3% and 10-year survival of 60-70%)^18–20^ or revision free survival of implant (5-year survival 71% and 10-year survival 63.3 %)^21^, some also report patient survival at various time points.^22^ Studies focusing on survival after resection and limb salvage of primary bone tumor in adults reported 2-year survival of 77%^16^, 3-year survival of 45.6 – 66.5% and 5-year survival of 38-67%^23–26^, with 5-year survival of limb salvage tumor operation around the knee in children of 72.7%.^27^ In this respect, our results show comparable patient survival rates for primary malignant bone tumors at 2 and 5 years.

Survival of patients with skeletal metastases is considerably shorter. Previously reported survival of proximal femur metastatic disease is 60% at 6 months and 35% at 12 months^28^, but the choice of treatment was significantly biased by initial stage of oncological disease. A recent study reported overall patient survival of 40% at 2 years and 28% at 4 years, no difference in survival between patient with solitary- or oligometastatic disease, and significantly better survival in comparison to multiple metastatic disease^29^, whereby the tumor diagnosis had considerable influence on the outcome of surgical metastasis treatment.^30^ Mean survival after modular endoprosthetic fixation was 860 days compared to 360 days after intramedullary bone fixation, showing statistically significant difference and higher complication rates of endoprosthetic reconstruction.^31^ A multicentric study reported mean survival of humerus metastasis of 16.7 months, significantly impacted by the occurrence of fracture, diaphyseal location and type of primary cancer^32^, while mean survival of pathological fractures of the humerus is reported as low as 8.3 months with 57 out of 87 cases treated by intramedullary nailing.^33^

Sørensen *et. al*. implemented 2013-SPRING model for prediction of survival after surgical treatment of bone metastases providing increased quality of life for patients while minimizing potential implant failure – 6-month postoperative survival was considered an indication for more durable implant and wider resection of metastatic lesion, because it cannot be expected for lesion to heal and internal fixation would likely lead to failure of implant.^5^ Similar findings were reported by Errani *et. al*. advocating that a postoperative survival of 12 months or more should include treatment with a more durable implant, whereby prognosis can be on just two parameters: histopathological diagnosis of metastases and elevated CRP values.^34^ In accordance with these previous findings, CRP was a reliable prognostic factor of shorter postoperative patient survival of metastatic patients in the presented study.

The presented study has several limitations. Data analysis only considered oncological patients with resected skeletal metastases and endoprosthetic reconstruction, while ignoring resections of spinal metastases and patients with intralesional metastatic tissue removal and palliative non-endoprosthetic stabilizations of long bones. Heterogeneity of patients in terms of age, gender, diagnoses, anatomical localizations, adjuvant radiotherapy or systemic therapy was another major limitation, likewise present in most similar studies on skeletal metastases. The confounding effect of added systemic therapy and/or radiotherapy was not controlled in our study. Nevertheless, the impact of these confounding factors was mitigated by using nation-wide uniform oncological guidelines and setting indications for oncological wide resections at a single oncological decision-making institution.

This is the first nation-wide cohort study to evaluate postoperative survival of patients after wide resection of skeletal metastases, treated at a single oncological institution. Patients with resected skeletal metastases had significantly shorter postoperative survival in comparison to primary malignant bone tumors or non-oncological revisions, but in most cases their survival was long enough to justify endoprosthetic reconstruction instead of less reliable palliative surgical solutions. Higher age, metastases other than plasmacytoma/renal cell/breast carcinoma, pathologic fracture or >1 metastasis should be considered relative contraindications for extensive resections and reconstructions.
